# Unlocking the potential of telehealth in Africa for HIV: opportunities, challenges, and pathways to equitable healthcare delivery

**DOI:** 10.3389/fdgth.2024.1278223

**Published:** 2024-03-04

**Authors:** Diego F. Cuadros, Qian Huang, Thulile Mathenjwa, Dickman Gareta, Chayanika Devi, Godfrey Musuka

**Affiliations:** ^1^ Digital Epidemiology Laboratory, Digital Futures, University of Cincinnati, Cincinnati, OH, United States; ^2^ Center for Rural Health Research, College of Public Health, East Tennessee State University, Johnson City, TN, United States; ^3^ Africa Health Research Institute, KwaZulu-Natal, Durban, South Africa; ^4^ International Initiative for Impact Evaluation, Harare, Zimbabwe

**Keywords:** telehealth, HIV, Africa, UNAIDS 95-95-95, digital barrier

## Introduction

Telehealth, using digital technology for remote healthcare services, offers transformative possibilities, especially for underserved regions. The growth of technology and connectivity promotes access to quality healthcare, mitigates healthcare disparities, and conquers geographic obstacles, connecting patients and providers irrespective of location. Telehealth provides an opportunity to ensure continuous care through remote monitoring and virtual consultations, improves health outcomes, and supports collaboration among healthcare professionals, extending services to deprived areas. Its innovative approach counteracts the scarcity of healthcare providers, enhances prompt and appropriate care, reduces patients' travel times, minimizes hospital stays, and facilitates healthcare access from home. This patient-centered approach not only saves valuable resources but places individuals at the core of the healthcare experience.

Some studies have confirmed the capacity of telehealth to provide primary healthcare services on par with traditional methods across various domains, showcasing its potential to address healthcare disparities (1, 2). However, recent concerns highlight worsening health disparities due to telehealth, especially amid the COVID-19 pandemic (3, 4). The expansion of digital health services is impeded by preexisting barriers, such as limited high-speed internet access in underprivileged communities, known as digital deserts (5). Proactive engagement and health equity policy implementation could prevent exacerbating healthcare disparities. In particular, the implementation of telehealth in low-income settings like Africa demands comprehensive exploration. This includes identifying health challenges telehealth can address, understanding adoption barriers recognizing benefiting or potentially excluded communities, and anticipating future challenges. By doing so, the practical and fair application of telehealth can enhance healthcare access and outcomes for African populations.

This opinion article explores the potential role of telehealth in Africa, particularly in tackling the HIV epidemic, and achieving the UNAIDS 95-95-95 goals, in which 95% of the people living with HIV know their status; of these, 95% are under an antiretroviral therapy (ART) regime, and form these under ART, 95% achieve viral load suppression. Barriers to implementation among patients, healthcare professionals, sectors, government and institutions, and the digital divide are discussed. Further, it explores key considerations and future challenges, aiming to successfully integrate telehealth and healthcare equity in Africa. This comprehensive exploration promotes equitable healthcare access and improves health outcomes for diverse African communities.

## Implementation of telehealth in the African context: barriers to telehealth implementation and digital divide

Telehealth has immense potential to overcome healthcare challenges, such as geographic obstacles, inadequate infrastructure, and limited access to services. It brings healthcare to remote, underserved regions, particularly low-income areas, using digital technology for remote consultations, diagnoses, treatments, and monitoring (6, 7). While telehealth has the capacity to enhance public health outcomes and mitigate disparities in Africa, there are still barriers in its implementation among patients, health professionals, healthcare sectors, and governments and institutions (8).

Telehealth faces adoption challenges in Africa due to patient resistance to change, economic and financial barriers, and privacy fears (9). Inherent habits, unawareness, or technophobia often hinder the shift from traditional in-person visits to digital healthcare (10). Socioeconomic barriers, especially in rural areas with limited Information and Communication Technology (ICT) infrastructure, electricity, and internet access, further limit its uptake. Concerns over medical data security and high costs associated with services and equipment compound these challenges (7).

Healthcare professionals in Africa face hurdles in telehealth adoption due to change resistance, skill and knowledge gaps, and fears of professional autonomy loss. Perceived threats to traditional healthcare benefits and roles can create hesitancy. Moreover, socio-cultural differences and language barriers sometimes favor in-person over virtual consultations, deeming them more effective (10, 11).

Financial, infrastructural, and operational constraints hinder telehealth adoption at the sectoral level. The high setup and maintenance costs, inadequate ICT infrastructure, and operational difficulties, such as the integration of telehealth into existing workflows, pose significant challenges (12). Countries like Zambia, Rwanda, Uganda, and Kenya grapple with issues tied to readiness of medical equipment, training, and software availability (13–15).

At the governmental and institutional level, limited political commitment, unsuitable policy frameworks, and technological constraints limit progress. Challenges encompass insufficient support, absent national telehealth policies, and gaps in e-health regulations. Infrastructure and technology shortfalls, along with usability issues with telehealth systems, further impede progress (16, 17).

The trajectory of internet utilization across the African continent has undergone a rapid and noteworthy expansion. Data from 2022 indicated that internet users on the continent have surpassed 570 million, more than doubling the figure reported in 2015 (18). This proliferation of internet access was largely attributable to enhancements in telecommunication infrastructure and the pervasive adoption of mobile technology. Nevertheless, the continent has not yet optimized its digital capabilities. As of 2021, the internet penetration rate stood at approximately 43%, which is substantially lower than the global average of 66% (19).

**Figure 1:**
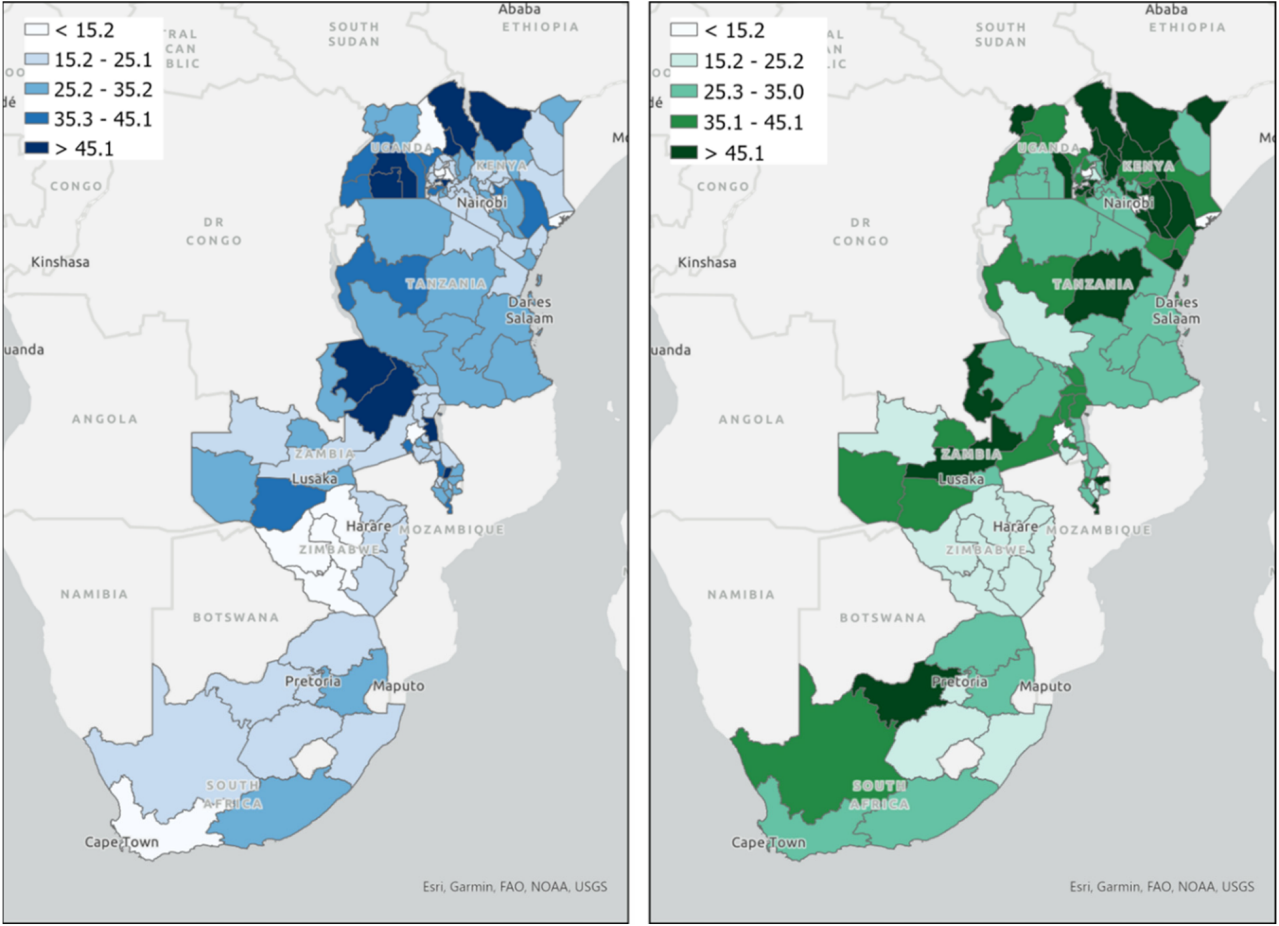
Spatial mapping of the percentage of internet usage for women and men in Eastern and Southern Africa. Maps illustrate the percentage of individuals that used internet at least once a week for women (map on the left) and men (map on the right). Maps were created using the most recent data from Demographic and Health surveys (20, 21) conducted in these countries between 2016 and 2021.

To illustrate the infrastructure barriers faced by African countries for telehealth implementation, internet use in some eastern and southern African countries, including Uganda, Kenya, Tanzania, Zambia, Malawi, Zimbabwe, and South Africa were mapped using the most recent data from Demographic and Health surveys (DHS), conducted between 2016 and 2021 (Figure 1). Briefly, the DHS is a cross-sectional household survey designed to collect nationally representative data on population, health, and socioeconomic variables (20). It is a major data source in low- and middle-income countries for a wide range of population and health indicators (20). They provide valuable insights into areas such as fertility, mortality, family planning, maternal and child health, nutrition, malaria, and HIV/AIDS (21). A detailed description of the surveys and the dataset can be found elsewhere (https://dhsprogram.com/) (20, 21). Maps illustrate a stark variation on internet usage among countries in general and for male and females. Urban areas, particularly capital cities and major economic hubs, tend to have higher internet penetration rates compared to rural locales. This disparity is often attributed to better infrastructure, greater availability of services, and higher income levels in urban centers. Rural areas, on the other hand, may face challenges such as lack of infrastructure, lower socioeconomic status, and limited access to digital literacy resources, leading to lower internet usage rates.

When comparing among the countries mapped, Kenya, South Africa and Zambia stand out with notably high internet penetration, reflecting its more developed infrastructure and economy relative to the other countries in this list. Conversely, nations like Malawi, Uganda and Zimbabwe have comparatively lower rates, indicating challenges in widespread internet accessibility. Such disparities are not only indicative of the state of digital infrastructure but also provide insights into broader developmental differences among these countries. The patterns suggest that while some countries have made significant strides in digital inclusion, others still have substantial ground to cover to bridge the digital divide. For males, regions such as Kenya, South Africa, and certain parts of Tanzania and Zambia show a higher concentration of internet users, as indicated by the darker shades of green on the map (map on the right in Figure 1). These areas indicate internet usage rates that exceed 45%. On the contrary, females in these countries generally exhibit lower internet usage rates (map on the left in Figure 1). While certain regions like Nairobi in Kenya or Pretoria in South Africa might exhibit relatively higher internet penetration among females, the overall distribution paints a picture of a wider gender-based digital divide (22).

The general patterns observed in internet usage across these regions can be attributed to a combination of socio-cultural, economic, and infrastructural factors. Internet penetration is growing in Sub-Saharan Africa. However, there remains a clear gender gap. Males often have greater access to the internet, influenced by socio-cultural norms that might restrict women's access to technology or prioritize male education and job opportunities in tech-related fields. Economic barriers further widen the gap, with the cost of devices and data potentially being prohibitive for many women (23). Lack of representation of women in tech-related jobs and top management positions in the information and communications technology sector is also a contributing factor (22).

Moreover, the infrastructure supporting internet and telecommunications in Africa, despite incremental advancements, remains comparatively underdeveloped, primarily due to inadequate investment (24). Compared to other global regions, the coverage of fiber networks in Africa is limited. In 2020, only 56% of the population in Sub-Saharan Africa resided within a 25-kilometer radius of an operational fiber optic network node. In the same timeframe, 4G technology was available to roughly 62% of African households, with regional penetration rates varying from about 60% in West and East Africa to nearly 90% in Northern Africa (18, 25). The expansion of mobile broadband coverage is a critical challenge in a continent where the majority of internet traffic is mobilized through mobile devices (18).

Given that telehealth relies heavily on consistent and reliable internet access, regions, especially in rural areas, with lower female internet usage might see reduced benefits from such services for women, who are already marginalized in many healthcare settings. The gender-based digital divide can potentially exacerbate existing health disparities, with women in areas of low internet penetration missing out on vital telehealth consultations, information dissemination, and remote medical services. Given the significant burden of HIV in many of these regions, telehealth has the potential to revolutionize care delivery, offering remote consultations, medication adherence monitoring, and patient education. However, with the observed lower internet access among females, there is a risk that a significant portion of the female population, already vulnerable to HIV due to various socio-economic factors, might miss out on these advanced care opportunities. This is especially concerning as women often play a central role in family health, and ensuring their access to telehealth can have multiplier effects on communities. Women in regions with low internet connectivity might face challenges in accessing virtual HIV counseling, teleconsultations for antiretroviral therapy, or even digital platforms that provide information on prevention and care (26).

For telehealth to be effectively integrated and benefit the entire population, it is imperative that initiatives addressing the gender digital gap are prioritized. Strategies could include increasing digital literacy programs for women, subsidizing internet access in low-connectivity regions, and actively promoting female participation in tech sectors, ensuring that telehealth's potential is realized across the entire demographic spectrum. Addressing this digital divide is crucial, not just for general health but to ensure that the strides made in HIV care reach all segments of the population, irrespective of gender or location. Ensuring equitable access to telehealth services in the context of HIV care would be critical in achieving broader public health goals in these countries.

Beyond the gender digital gap, the digital divide between rural and urban areas in Africa is a pressing issue that significantly impacts the efficacy of telehealth initiatives. With nearly 60% of Africa's population residing in rural areas (27), where internet penetration is drastically lower at 23%, compared to 64% in urban locales (28), the challenge is not only to improve healthcare access but also to bridge this technological chasm. To reduce this gap, a multipronged approach is required. Investment in infrastructure is critical; governments, with the support of international partnerships, must prioritize the expansion of broadband connectivity into rural areas. Additionally, low-cost, low-bandwidth solutions must be developed to make telehealth services viable where connectivity remains limited (29). Innovative use of widely available technologies, such as basic mobile phones and radio, can play an important role in reaching out to remote populations. For instance, SMS-based health tips and appointment reminders can be effective in areas where smartphones and internet access are scarce (30, 31). Furthermore, community-based interventions, like establishing local telehealth kiosks with internet access, can serve as intermediary points for healthcare delivery (32). Training and employing local community health workers to leverage these technologies can also help in mitigating the divide (29). These workers can act as a bridge between the digital healthcare platforms and the local populace, guiding them in the use of telehealth services. Lastly, policy frameworks must be adapted to encourage and facilitate private-sector investment in rural internet infrastructure, ensuring that the expansion of digital services is inclusive and equitable (33).

## Leveraging telehealth for HIV prevention, care, and support in Africa: advantages and challenges

The relentless HIV epidemic in Africa, particularly in rural areas with a dense disease burden, demands urgent attention and innovative interventions. Housing approximately two-thirds of the global HIV populace, Africa grapples with persistent challenges in curtailing the epidemic, despite overall progress in minimizing new infections (34). The potential solution to this lies in adopting modern strategies like telehealth, with the objective of understanding its benefits and challenges in preventing and managing HIV and providing support in the African milieu.

The rise of telehealth tools in the arena of HIV prevention and care across Africa has been a central point in contemporary studies (see references in Supplementary Table S1). Mobile devices have emerged as central instruments, bolstering self-management, psychological well-being, and active participation, particularly among undisclosed HIV-positive youth, thus broadening the scope of care. Furthermore, telehealth initiatives have demonstrated their efficacy in catalyzing HIV testing among at-risk groups, though there is a call for more targeted strategies. Notwithstanding hurdles like crafting theoretical models and safeguarding patient privacy, telehealth methodologies are perceived as viable and well-received among marginalized HIV-afflicted individuals with concurrent substance abuse issues.

Telehealth leverages digital tools to enhance access to HIV prevention, testing, care, and support services, particularly for individuals living with or vulnerable to HIV. It holds the potential to transform HIV care by addressing challenges like geographical distance and scarce healthcare infrastructure, especially in remote and underserved areas where conventional healthcare access is deficient. Telehealth also enhances retention in care, which is crucial for effective treatment outcomes. It facilitates virtual consultations, remote monitoring, and follow-ups by mitigating barriers like transport costs, waiting times, and stigma associated with clinic visits. This ensures better adherence to antiretroviral therapy (ART), access to support services, and ultimately improved health outcomes. Furthermore, telehealth provides timely interventions in HIV prevention and care, including virtual counseling, behavior change interventions, and medication adherence support, tailored to individual needs. These advantages of telehealth implementation would be paramount for the achievement of The Joint United Nations Programme on HIV/AIDS (UNAIDS) 95-95-95 targets, and for the sustainability of the HIV care in a post UNAIDS targets era.

The tumultuous times during the COVID-19 pandemic offered a prism into the multifaceted challenges and advantages of telehealth in crisis scenarios. In the wake of the COVID-19 pandemic, HIV/AIDS programs in resource-constrained nations faced heightened challenges. The unprecedented nature of the pandemic necessitated innovative strategies to sustain HIV prevention and treatment, particularly for key demographics and voluntary medical male circumcision (VMMC) services in the 15 sub-Saharan African nations prioritized by the US President's Emergency Plan for AIDS Relief (35). A key response was the expanded and diversified application of telehealth, a transformation catalyzed by the pandemic, which enhanced delivery of healthcare accessibility, flexibility, and efficiency (36).

For instance, during this period, Kenya amplified their outreach through calls, texts, and videos, partnering with digital influencers to increase Pre-Exposure Prophylaxis (PrEP) awareness among adolescent girls and young women (AGYW). In high-HIV-burden countries, there was a marked rise in leveraging apps and video calls to link HIV peer influencers with their clientele. Digital platforms, now integral for scheduling HIV prevention services and procuring ART refills, have demonstrated their impact. Under Kenya's EpIC initiative, online HIV case findings post-pandemic surged to 10% from a mere 5% pre-COVID-19, indicating the enduring potential of such digital tools (37).

Countries like Botswana, Eswatini, Rwanda, Tanzania, and Zimbabwe have commendably achieved the 95-95-95 UNAIDS benchmarks, with at least 16 other nations, including eight in sub-Saharan Africa, on the cusp of this accomplishment (37). These milestones pave the way for broader application of telehealth in enhancing HIV-related healthcare delivery. A paramount consideration is ensuring that people living with HIV (PLHIV) on treatment remain adherent and are not lost to follow-ups. Telehealth can facilitate seamless access to patient records, ensuring efficient transitions between health facilities. Moreover, as the PLHIV demographic ages, non-communicable diseases (NCDs) like cancers, diabetes, and hypertension gain prominence (38). With ART patients also on NCD medications, potential drug interactions pose risks. These interactions can compromise ART adherence, elevating treatment failure risks and necessitating advanced ART treatments. To safeguard the strides made in HIV response, it is crucial that PLHIV, especially in remote areas, can consult specialist physicians to address ART treatment challenges and drug interactions. Furthermore, the introduction of long-acting extended delivery (LAED) formulations like injectable cabotegravir (CAB-LA) and dapivirine vaginal ring (DPV-VR) has enriched the HIV prevention arsenal. These are especially critical for high-risk groups like AGYW (37). However, as the deployment of these formulations escalates in developing nations, potential side effects could arise, necessitating specialist consultations even in remote areas with the use of telehealth.

Numerous studies have focused on assessing the advantages of telehealth in the realm of HIV services and patient management (see references in Supplementary Table S1). One such study accentuated the potency of telehealth, notably through remote videoconferencing, as a central tool in counseling HIV-positive African American youth, highlighting its convenience and transformative potential in bolstering medication adherence (39). Other studies have showcased the transformative potential of distant consultations and mentorship for common HIV providers (40, 41). Medical professionals have acknowledged the dual-edged nature of telemedicine, underscoring the imperative for expansive education (42). Subsequent studies spotlighted the instrumental role of telehealth in fortifying consistent viral suppression, coupled with its promise in augmenting HIV care engagement, especially among the youth demographic (43, 44). Delving into patient perspectives in subsequent research, telemedicine emerged with a spectrum of benefits tempered by concerns, predominantly centered around technological facets and privacy considerations (45–47). Concurrently, research emanating from Vietnam illustrated the capacity of telehealth to fortify the competencies of healthcare professionals in HIV care (41).

In the South African landscape, the concept of mobile-driven HIV screening has been championed as a cost-efficient alternative with the potential to elevate survival outcomes. The transformative power of telehealth campaigns in championing patient autonomy, especially for those in the HIV risk bracket, has been accentuated. Additionally, the exploration into mobile applications designed to relay HIV test outcomes and fortify the bridge to post-diagnosis HIV care has been undertaken, with an emphasis on patient narratives.

Supplementary Table S1 lists and summarizes studies mentioned above. The advantages of telehealth for HIV prevention, care, and support in Africa include:

• *Health Education and Behavioral Change*: Telehealth platforms can deliver targeted health education and behavior change interventions to promote safer practices and reduce the risk of HIV transmission. Personalized text messages, videos, and mobile apps can be utilized to provide information, support, and reminders to individuals at risk or living with HIV, helping to achieve the behavioral change necessary for reaching the 95-95-95 UNAIDS targets (40, 48).
• *Enhanced Access to HIV Services*: Telehealth transcends geographical barriers, enabling the provision of healthcare services to individuals residing in remote or underserved areas with limited access to conventional healthcare facilities. This aspect assumes paramount importance in Africa, where the concentration of the HIV epidemic is observed in rural regions. Consequently, telehealth has the potential to be transformative, effectively bridging the gap and bringing essential services closer to the populations most in need, particularly during the 95-95-95 UNAIDS targets era (49–51).
• *HIV Testing and Diagnosis*: Telehealth can facilitate widespread HIV testing and diagnosis by enabling remote testing services and delivering test results electronically. This approach can help reach individuals at higher risk of acquiring HIV, men, and those less likely to use health facilities or community-based services. By ensuring that 95% of people living with HIV know their HIV status, the first UNAIDS targets can be achieved (51, 52).
• *ART Adherence*: Telehealth platforms can provide support and reminders for individuals on ART, helping them adhere to their medication regimens. Remote monitoring of treatment adherence and viral suppression can be facilitated through telehealth tools, ensuring that 95% of diagnosed individuals receive sustained antiretroviral therapy (53, 54).
• *Viral Suppression Monitoring*: Telehealth can enable remote monitoring of viral suppression among individuals receiving antiretroviral therapy. Regular virtual consultations and remote laboratory monitoring can help ensure that 95% of individuals on ART achieve viral suppression (43, 44).
• *Patient Follow-up and Care Management*: Telehealth enables remote patient follow-up and care management, reducing the need for in-person visits and improving access to care. Virtual consultations, remote monitoring of symptoms and side effects, and medication adherence support can all be delivered through telehealth platforms, ensuring continuity of care and enhancing the overall healthcare experience for individuals living with HIV (47).
• *Peer Support and Education*: Telehealth platforms provide a conducive environment for facilitating virtual peer support groups and educational sessions, creating a sense of community among individuals with HIV. These virtual networks foster psychosocial support, diminish feelings of isolation, and promote adherence to HIV treatment regimens. Peer-led discussions, experiential sharing, and accurate information dissemination empower individuals and strengthen their engagement in care (40).

Zooming out, numerous investigations underscore the promise of mobile innovations in refining HIV care, spanning from amplifying awareness and facilitating tests to ensuring consistent treatment adherence. Nevertheless, there is a shared sentiment underscoring the imperative for robust, evidence-driven research to corroborate the advantages and challenges and navigate the intricacies of these interventions across the diverse African milieu.

The implementation of telehealth for HIV care in Africa presents several barriers, despite its potential benefits. Key challenges include limited access to mobile devices and internet connectivity, especially in rural and resource-limited settings. Socio-demographic disparities, such as gender differences in mobile device usage, further exacerbate these accessibility issues. Confidentiality concerns arise, given the sensitive nature of HIV status and the potential for breaches in patient data through telehealth platforms. Additionally, while telehealth interventions can be feasible and acceptable, there is a need for more tailored and rigorous strategies to address the unique needs of marginalized populations, including non-disclosed youth living with HIV and those with substance use issues. The lack of established theoretical frameworks for telehealth interventions and the nascent state of these technologies in the African context also pose challenges. Furthermore, while mobile HIV screening and telehealth campaigns hold promise, there is a call for evidence-based research to validate their benefits and ensure effective integration into the broader healthcare system.

More specifically, implementing telehealth for HIV in Africa also faces some of the following challenges:

• *Patients:* The major obstacles of HIV patients in Africa are primarily related to technology access, and privacy and confidentiality. Many patients lack the necessary digital devices or reliable internet access. To ensure patient information's secure transmission and storage, telehealth platforms must adhere to robust data protection measures and comply with relevant regulations and guidelines. Addressing privacy concerns, implementing encryption protocols, and establishing trustworthy systems are vital to maintaining patient trust and safeguarding the confidentiality of telehealth services (50, 55, 56).
• *Health Professionals:* The effective delivery of HIV services through telehealth requires healthcare providers to receive adequate training. This includes acquiring proficiency in telehealth technologies, honing virtual counseling skills, and developing strategies to maintain patient engagement within a remote setting. Building the capacity of healthcare providers is indispensable for ensuring the delivery of high-quality, culturally competent care through telehealth platforms (41, 55).
• *Health Sectors:* The implementation of telehealth puts a strain on already limited resources. The maintenance of telehealth systems for HIV services can be costly and may lack technical support. Effective interoperability of health systems is needed to coordinate care between telehealth and traditional health services (56, 57).
• *Government and Institutions*: Maximizing telehealth interventions' impact on HIV in Africa necessitates a comprehensive approach addressing equity and inclusivity issues. Legal and regulatory challenges exist among governments and institutions. Overcoming language barriers and cultural sensitivities, and catering to the specific needs of marginalized populations, including women, adolescents, and key populations at a higher risk of HIV infection, are imperative. Ensuring the accessibility, acceptability, and effectiveness of telehealth services for all individuals, irrespective of their background or circumstances, is paramount (57).

While telehealth holds promise in managing the HIV epidemic in Africa, challenges like the digital divide, privacy concerns, training needs, and inclusion issues must be addressed. If tackled effectively, telehealth can enhance existing healthcare systems, contributing significantly to HIV prevention, care, and support.

## Discussion

In summary, telehealth holds transformative potential for healthcare in Africa, especially in underprivileged regions, presenting a decisive tool for attaining the UNAIDS 95-95-95 goals by 2030, and for ensuring a sustainable HIV care during a post UNAIDS targets era. By harnessing digital innovations, telehealth is poised to bridge healthcare disparities, tackle specific health issues, and boost access to high-quality care. Nevertheless, realizing the full capacity of telehealth, particularly in managing the HIV epidemic, requires overcoming implementation obstacles and fostering stakeholder collaboration. With sustained commitment and investments, telehealth could significantly reshape healthcare outcomes on the continent.

Telehealth's proliferation in Africa brings both prospects and challenges. Initiatives aimed at capacity-building and training are crucial in equipping health professionals to utilize telehealth for quality remote care delivery adeptly. Symbiotic relations between governments, health institutions, and tech providers are key to fostering a robust telehealth infrastructure and formulating policies that support its execution. Moreover, addressing regulatory parameters, data privacy issues, and reimbursement models is essential for the successful implementation of telehealth in the African context. As the field evolves, relentless evaluation of its impact, advocating research and innovation, and strategy refinement are imperative to navigate implementation challenges for HIV care.

Looking forward, the concerted efforts of the government, private sector, and communities will be decisive in advancing the adoption of telehealth in HIV prevention and care in Africa. The government's infrastructural investments, training initiatives, and policy support can significantly aid this process. The private sector can propel this advancement by developing telehealth services, investing in infrastructure, and promoting telehealth usage among providers and patients. Communities, through raising awareness of telehealth's merits, patient education, and backing telehealth initiatives, can impact this evolution. Through this collaborative framework, telehealth can become an integral part of the HIV healthcare mosaic in Africa, enhancing quality care access and eventually revolutionizing healthcare outcomes in the region.
