# Supplementary material for: Unlocking the potential of telehealth in Africa for HIV: opportunities, challenges, and pathways to equitable healthcare delivery
Source: Front Digit Health. 2024 Mar 4;6:1278223. doi: 10.3389/fdgth.2024.1278223 (PMC10944905; doi:10.3389/fdgth.2024.1278223)
Supplement: Supplementary file 1 [file Table1.pdf]

SUPPLEMENTARY MATERIALS FOR:

Unlocking the Potential of Telehealth in Africa for HIV: Opportunities, Challenges, and Pathways to Equitable Healthcare Delivery

Diego F Cuadros[1], Qian Huang[2], Thulile Mathenjwa[3], Dickman Gareta[3], Chayanika Devi[1], Godfrey Musuka[4]

[1] Digital Epidemiology Laboratory, Digital Futures, University of Cincinnati, Cincinnati, OH, USA

[2] Center for Rural Health Research, College of Public Health, East Tennessee State University, Johnson City, TN, USA

[3] Africa Health Research Institute, KwaZulu-Natal, Durban, South Africa

[4] International Initiative for Impact Evaluation, Harare, Zimbabwe

Supplementary Table 1

Title	Year	Summary
**Section A – Telehealth in Africa**		
A Pilot Study to Engage and Counsel HIV-Positive African American Youth Via Telehealth Technology [1]	2013	This study assessed the feasibility and acceptability of a telehealth (remote videoconferencing) medication counseling intervention for HIV-positive African American youth. The participants found telehealth convenient and efficient, and it provided a modality to interact with providers that was less intimidating than in-person visits.
Mixed-Methods Evaluation of a Telehealth Collaborative Care Program for Persons with HIV Infection in a Rural Setting [2]	2013	The study discusses the challenges of delivering comprehensive care for persons with HIV in rural settings. A telehealth collaborative care program was developed and evaluated. The program integrated specialty care delivered via clinical video telehealth with primary care from generalist providers in seven Community Based Outpatient Clinics. The study found that the telehealth collaborative care approach is feasible for providing accessible care in rural settings.
Geographic Access and Use of Infectious Diseases Specialty and General Primary Care Services by Veterans with HIV Infection: Implications for Telehealth and Shared Care Programs [3]	2014	The study explores the geographic access and use of infectious disease specialty and general primary care services by veterans with HIV. It highlights the challenges faced by rural-dwelling persons with HIV in accessing specialty care and suggests that telehealth can be a solution to deliver care over distance and establish "shared care" relationships with distant providers.
Impact of a Telehealth Program That Delivers Remote Consultation and Longitudinal Mentorship to Community HIV Providers [4]	2016	The study discusses the implementation and outcomes of a telehealth program designed to enhance HIV care capacity in various regions (ECHO). The study found that the ECHO telementoring method was effective in delivering clinical education and support. It highlighted the potential of such telehealth interventions to expand HIV care capacity across vast geographic regions, ensuring that patients receive timely and appropriate care regardless of their location.
Physician's Perceptions of Telemedicine in HIV Care Provision: A Cross-Sectional Web-Based Survey [5]	2017	This study explored the views of physicians regarding the use of telemedicine in the care of patients living with HIV. The research aimed to understand how doctors perceive the potential benefits and challenges of integrating telemedicine into HIV care. The findings suggest that telemedicine is seen as a promising tool in the care of patients with HIV, indicating its potential to enhance patient care and improve health outcomes for this specific patient population
Impact of Availability of Telehealth Programs on Documented HIV Viral Suppression: A Cluster-Randomized Program Evaluation in the Veterans Health Administration. [6]	2019	This study was conducted to assess the effects of telehealth programs on HIV viral suppression, specifically within the Veterans Health Administration. The study underscores the potential of telehealth as a viable tool in managing and improving the health of individuals living with HIV/AIDS, particularly in ensuring consistent viral suppression.
Telehealth and texting intervention to improve HIV care engagement, mental health and substance use outcomes in youth living with HIV: a pilot feasibility and acceptability study protocol [7]	2019	The study underscores the potential of telehealth as a viable tool in managing and improving the health of individuals living with HIV/AIDS, particularly in ensuring consistent viral suppression.
Exploring the Attitude of Patients with HIV About Using Telehealth for HIV Care [8]	2020	This study explores the attitudes of people with HIV (PWH) about using telemedicine for care instead of face-to-face clinic visits. The study found that telehealth programs can improve HIV care but concerns about technology and privacy need to be addressed to increase acceptability and usage among PWH.
Building HIV healthcare worker capacity through telehealth in Vietnam [9]	2020	This study discusses the utilization of telehealth as a tool to enhance the capacity of healthcare workers in Vietnam, specifically those involved in HIV care.<br>The study draws insights from Vietnam's experience with telehealth and emphasizes its potential in offering a sustainable approach to HIV technical assistance and health worker capacity building.
Effects of telehealth-assisted interventions among people living with HIV/AIDS: A systematic review and meta-analysis of randomized controlled studies. [10]	2021	This study suggested that delivering health management interventions remotely through telehealth-assisted modalities was both feasible and effective in yielding health benefits for people living with HIV/AIDS.
Telemedicine for HIV Care: Current Status and Future Prospects [11]	2021	This study emphasizes the need for future research on the provision of HIV care using telemedicine beyond the pandemic. It also suggests focusing on ways to improve telemedicine services for HIV care.

Employing telehealth within HIV care: advantages, challenges, and recommendations [12]	2021	This article discusses the key advantages of employing telehealth in HIV care. One significant benefit is the reduction of stigma-related delays in care, as many people with HIV report high levels of stigma.
Experiences with Telemedicine for HIV Care During the COVID-19 Pandemic: A Mixed-Methods Study [13]	2022	This study aimed to understand the perspectives and experiences of individuals with telemedicine during the COVID-19 pandemic, specifically in the context of HIV care. The researchers conducted a mixed-methods study in two HIV clinics in the US. The study explored the benefits, challenges, and overall effectiveness of telemedicine in ensuring continuous care for HIV patients during such unprecedented times and emphasizes the need to optimize telemedicine for HIV care. It suggests strategies to improve technology support for people with HIV (PWH) and offers flexible options for accessing care.
HIV Care Meets Telehealth: a Review of Successes, Disparities, and Unresolved Challenges [14]	2022	This article reviews the successes and challenges of integrating telehealth into HIV care. They found that while telehealth remains a valuable tool in HIV care, there exist disparities in access and health outcomes. Factors such as telehealth literacy and broadband access might play a role in these disparities.
Does telehealth affect the adherence to ART among patients with HIV? A systematic review and meta-analysis[15]	2023	This article discusses the different effects of telehealth interventions on adherence to Antiretroviral therapy (ART) among people living with HIV.
**Section B – Telehealth implementation for HIV care in Africa**		
Mobile health applications for HIV prevention and care in Africa [16]	2015	This review identified applications of m-health for HIV prevention and care categorized by the following three themes: patient-care focused applications, such as health behavior change, health system-focused applications, such as reporting and data collection, and population health-focused applications, including HIV awareness and testing campaigns.
Impact of telemonitoring approaches on integrated HIV and TB diagnosis and treatment interventions in sub-Saharan Africa: a scoping review [17]	2017	This study explored telemonitoring and mHealth approaches as potential strategies in addressing HIV and TB. The emphasis was on real-time and contextual strategies that could revolutionize the treatment and management of these diseases
Use of mobile phones and text messaging to decrease the turnaround time for early infant HIV diagnosis and notification in rural Zambia: an observational study [18]	2017	This observational study aimed to assess the impact of mobile technology on improving the efficiency and timeliness of HIV diagnosis and notification for infants in resource-limited setting. Mobile phone and text messaging technology has the potential to improve early infant diagnosis but challenges to widespread implementation need to be addressed, including low mobile phone ownership, use and coverage in rural areas.
Integration of telehealth services in the healthcare system: with emphasis on the experience of patients living with HIV [19]	2019	This study emphasizes the need for well-designed studies to show that the implementation of telehealth could improve the HIV care continuum
Smartphone usage and preferences among postpartum HIV-positive women in South Africa [20]	2019	This study explored the acceptability and feasibility of mobile health (mHealth) interventions in the context of postpartum women living with HIV. Focus group discussions were conducted to gather insights and understand the potential of mHealth interventions for this demographic
Evaluation of the ELIMIKA Pilot Project: Improving ART Adherence among HIV Positive Youth Using an eHealth Intervention in Mombasa, Kenya [21]	2019	The study focused on the usability and effectiveness of a pilot digital peer support platform named "ELIMIKA" for HIV-positive youths in Mombasa, Kenya. The primary objective was to evaluate how this digital platform could aid in improving adherence to Antiretroviral Therapy (ART) and overall HIV care among the youth demographic.
Exploring People's Candidacy for Mobile Health-Supported HIV Testing and Care Services in Rural KwaZulu-Natal, South Africa: Qualitative Study [22]	2019	The study aimed to provide insights into the potential use of mobile health-supported services as a way to improve access to HIV testing and care services in rural areas. Although mHealth provides a way for individuals to test their candidacy for HIV services, the barriers that can make the service unattractive at the clinic level will also need to be addressed if potential demand is to turn into actual demand.
Computer-Based Counseling Program (CARE+ Kenya) to Promote Prevention and HIV Health for People Living With HIV/AIDS: A Randomized Controlled Trial [23]	2019	The study conducted in Kenya focused on evaluating a computer-based counseling tool designed for HIV prevention and adherence services. The study suggests that such computer-based interventions can play a crucial role in enhancing HIV prevention efforts and improving adherence to treatment, especially in regions with high HIV prevalence.
Cell Phone Counseling Improves Retention of Mothers With HIV Infection in Care and	2019	This study from Kenya focuses into the effectiveness of a structured cell phone counseling intervention aiming to promote the retention of HIV-positive mothers in care and encourage HIV testing in infants. the study showcased the potential of mobile-based interventions in enhancing care retention and promoting essential medical testing in vulnerable populations

Infant HIV Testing in Kisumu, Kenya: A Randomized Controlled Study [24]		
Patient and health-care worker experiences of an HIV viral load intervention using SMS: A qualitative study [25]	2019	This study focused on the use of SMS (Short Message Service) as an intervention to manage and communicate viral load (VL) results to patients living with HIV in rural districts of Zimbabwe. The findings highlighted the potential of digital health interventions, like SMS, to bridge the gap in healthcare communication, especially in remote areas where traditional methods might be less efficient or timely.
Formative research for an mHealth program to improve the HIV care continuum in South Africa [26]	2020	This study found that an mHealth program to deliver HIV test results directly to patients could mitigate multiple barriers to care but needs to be tested for efficacy. Concerns identified by patients and providers must be addressed in designing the program to successfully integrate with health facility workflow and ensure its sustainability.
Mobile Device Usage by Gender Among High-Risk HIV Individuals in a Rural, Resource-Limited Setting [27]	2021	This study focused in the characterization of the use of mobile devices in rural KwaZulu-Natal, South Africa to tailor mHealth interventions for people living with HIV and at risk for acquiring HIV in the middle-income country. The study found that as women are more likely to own smartphones, smartphone-based mHealth interventions specifically geared to prevent the acquisition of or to support the care of HIV in young women in KwaZulu-Natal may be feasible.

References

[1] P. Saberi, P. Yuan, M. John, N. Sheon, and M.O. Johnson, A pilot study to engage and counsel HIV-positive African American youth via telehealth technology. AIDS Patient Care STDS 27 (2013) 529-32.

[2] M. Ohl, D. Dillon, J. Moeckli, S. Ono, N. Waterbury, J. Sissel, J. Yin, B. Neil, B. Wakefield, and P. Kaboli, Mixed-methods evaluation of a telehealth collaborative care program for persons with HIV infection in a rural setting. J Gen Intern Med 28 (2013) 1165-73.

[3] M.E. Ohl, K. Richardson, P.J. Kaboli, E.N. Perencevich, and M. Vaughan-Sarrazin, Geographic access and use of infectious diseases specialty and general primary care services by veterans with HIV infection: implications for telehealth and shared care programs. J Rural Health 30 (2014) 412-21.

[4] B.R. Wood, K.T. Unruh, N. Martinez-Paz, M. Annese, C.B. Ramers, R.D. Harrington, S. Dhanireddy, L. Kimmerly, J.D. Scott, and D.H. Spach, Impact of a Telehealth Program That Delivers Remote Consultation and Longitudinal Mentorship to Community HIV Providers. Open Forum Infect Dis 3 (2016) ofw123.

[5] K. Anderson, T. Francis, F. Ibanez-Carrasco, and J. Globerman, Physician's Perceptions of Telemedicine in HIV Care Provision: A Cross-Sectional Web-Based Survey. JMIR Public Health Surveill 3 (2017) e31.

[6] M.E. Ohl, K. Richardson, M.C. Rodriguez-Barradas, R. Bedimo, V. Marconi, J.P. Morano, M.P. Jones, and M. Vaughan-Sarrazin, Impact of Availability of Telehealth Programs on Documented HIV Viral Suppression: A Cluster-Randomized Program Evaluation in the Veterans Health Administration. Open Forum Infect Dis 6 (2019) ofz206.

[7] A.R. Wootton, D.A. Legnitto, V.A. Gruber, C. Dawson-Rose, T.B. Neilands, M.O. Johnson, and P. Saberi, Telehealth and texting intervention to improve HIV care engagement, mental health and substance use outcomes in youth living with HIV: a pilot feasibility and acceptability study protocol. BMJ Open 9 (2019) e028522.

[8] D. Dandachi, B.N. Dang, B. Lucari, M. Teti, and T.P. Giordano, Exploring the Attitude of Patients with HIV About Using Telehealth for HIV Care. AIDS Patient Care STDS 34 (2020) 166-172.

[9] T.M. Pollack, V.T.T. Nhung, D.T.N. Vinh, D.T. Hao, L.T.T. Trang, P.A. Duc, N.V. Kinh, N.T.H. Dung, D.L. Dung, N.T. Ninh, H.T.T. Huyen, V.X. Huy, D.M. Hai, T.H. Khanh, N.T.T. Hien, P.T.A. Khuong, N.T. Trong, N.V. Lam, V.N. Phinh, D.T. Phuong, N.D. Duat, N.T. Liem, N.T. Binh, N.K. Chi, L.N. Yen, and L. Cosimi, Building HIV healthcare worker capacity through telehealth in Vietnam. BMJ Glob Health 5 (2020) e002166.

[10] I.D. Saragih, S.I. Tonapa, C.P. Osingada, C.M. Porta, and B.O. Lee, Effects of telehealth-assisted interventions among people living with HIV/AIDS: A systematic review and meta-analysis of randomized controlled studies. J Telemed Telecare (2021) 1357633X211070726.

[11] E. Smith, and M.E. Badowski, Telemedicine for HIV Care: Current Status and Future Prospects. HIV AIDS (Auckl) 13 (2021) 651-656.

[12] M. Grove, L.L. Brown, H.K. Knudsen, E.G. Martin, and B.R. Garner, Employing telehealth within HIV care: advantages, challenges, and recommendations. AIDS 35 (2021) 1328-1330.

[13] D. Harsono, Y. Deng, S. Chung, L.A. Barakat, G. Friedland, J.P. Meyer, E. Porter, M. Villanueva, M.S. Wolf, J.E. Yager, and E.J. Edelman, Experiences with Telemedicine for HIV Care During the COVID-19 Pandemic: A Mixed-Methods Study. AIDS Behav 26 (2022) 2099-2111.

[14] T. Labisi, N. Regan, P. Davis, and N. Fadul, HIV Care Meets Telehealth: a Review of Successes, Disparities, and Unresolved Challenges. Curr HIV/AIDS Rep 19 (2022) 446-453.

[15] E.D. Esmaeili, H. Azizi, S. Dastgiri, and L.R. Kalankesh, Does telehealth affect the adherence to ART among patients with HIV? A systematic review and meta-analysis. BMC Infect Dis 23 (2023) 169.

[16] J.I. Forrest, M. Wiens, S. Kanters, S. Nsanzimana, R.T. Lester, and E.J. Mills, Mobile health applications for HIV prevention and care in Africa. Curr Opin HIV AIDS 10 (2015) 464-71.

[17] C.S. Yah, E. Tambo, C. Khayeka-Wandabwa, and J.Y. Ngogang, Impact of telemonitoring approaches on integrated HIV and TB diagnosis and treatment interventions in sub-Saharan Africa: a scoping review. Health Promot Perspect 7 (2017) 60-65.

[18] C.G. Sutcliffe, P.E. Thuma, J.H. van Dijk, K. Sinywimaanzi, S. Mweetwa, M. Hamahuwa, and W.J. Moss, Use of mobile phones and text messaging to decrease the turnaround time for early infant HIV diagnosis and notification in rural Zambia: an observational study. BMC Pediatr 17 (2017) 66.

[19] D. Dandachi, C. Lee, R.O. Morgan, S. Tavakoli-Tabasi, T.P. Giordano, and M.C. Rodriguez-Barradas, Integration of telehealth services in the healthcare system: with emphasis on the experience of patients living with HIV. J Investig Med 67 (2019) 815-820.

[20] P. Mogoba, T.K. Phillips, L. Myer, L. Ndlovu, M.C. Were, and K. Clouse, Smartphone usage and preferences among postpartum HIV-positive women in South Africa. AIDS Care 31 (2019) 723-729.

[21] O. Ivanova, S. Wambua, J. Mwaisaka, T. Bossier, M. Thiongo, K. Michielsen, and P. Gichangi, Evaluation of the ELIMIKA Pilot Project: Improving ART Adherence among HIV Positive Youth Using an eHealth Intervention in Mombasa, Kenya. Afr J Reprod Health 23 (2019) 100-110.

[22] O. Adeagbo, C. Herbst, A. Blandford, R. McKendry, C. Estcourt, J. Seeley, and M. Shahmanesh, Exploring People's Candidacy for Mobile Health-Supported HIV Testing and Care Services in Rural KwaZulu-Natal, South Africa: Qualitative Study. J Med Internet Res 21 (2019) e15681.

[23] A.E. Kurth, J.E. Sidle, N. Chhun, J.A. Lizcano, S.M. Macharia, M.M. Garcia, A. Mwangi, A. Keter, and A.M. Siika, Computer-Based Counseling Program (CARE+ Kenya) to Promote Prevention and HIV Health for People Living With HIV/AIDS: A Randomized Controlled Trial. AIDS Educ Prev 31 (2019) 395-406.

[24] A. Sarna, L.R. Saraswati, J. Okal, J. Matheka, D. Owuor, R.J. Singh, N. Reynolds, and S. Kalibala, Cell Phone Counseling Improves Retention of Mothers With HIV Infection in Care and Infant HIV Testing in Kisumu, Kenya: A Randomized Controlled Study. Glob Health Sci Pract 7 (2019) 171-188.

[25] E. Venables, Z. Ndlovu, D. Munyaradzi, G. Martinez-Perez, E. Mbofana, P. Nyika, H. Chidawanyika, D.B. Garone, and H. Bygrave, Patient and health-care worker experiences of an HIV viral load intervention using SMS: A qualitative study. PLoS One 14 (2019) e0215236.

[26] L. DiAndreth, N. Krishnan, J.L. Elf, S. Cox, C. Tilchin, M. Nthulana, B. Jarrett, N. Kronis, E. Dupuis, K. Motlhaoleng, S. Chon, N. Martinson, and J.E. Golub, Formative research for an mHealth program to improve the HIV care continuum in South Africa. AIDS Care 32 (2020) 744-748.

[27] K.C. Breanna R Campbell 1, Megan Gray Neils 1, Chelsea Canan 1, Anthony Moll 3, Rebecca Dillingham 1, Sheela V Shenoi 2, Mobile Device Usage by Gender Among High-Risk HIV Individuals in a Rural, Resource-Limited Setting. Telemed J E Health 27(6) (2021) 615-624.
